# Behavioural, Pharmacokinetic, Metabolic, and Hyperthermic Profile of 3,4-Methylenedioxypyrovalerone (MDPV) in the Wistar Rat

**DOI:** 10.3389/fpsyt.2018.00144

**Published:** 2018-04-24

**Authors:** Rachel R. Horsley, Eva Lhotkova, Katerina Hajkova, Barbara Feriancikova, Michal Himl, Martin Kuchar, Tomas Páleníček

**Affiliations:** ^1^Department of Experimental Neurobiology, National Institute of Mental Health, Klecany, Czechia; ^2^Forensic Laboratory of Biologically Active Compounds, Department of Chemistry of Natural Compounds, University of Chemistry and Technology, Prague, Czechia; ^3^Department of Analytical Chemistry, University of Chemistry and Technology, Prague, Czechia; ^4^Department of Organic Chemistry, University of Chemistry and Technology, Prague, Czechia

**Keywords:** 3 4-methylenedioxypyrovalerone, behaviour, hyperthermia, locomotion, MDPV, pharmacokinetics, sensory gating, wistar rat

## Abstract

3,4-methylenedioxypyrovalerone (MDPV) is a potent pyrovalerone cathinone that is substituted for amphetamines by recreational users. We report a comprehensive and detailed description of the effects of subcutaneous MDPV (1–4 mg/kg) on pharmacokinetics, biodistribution and metabolism, acute effects on thermoregulation under isolated and aggregated conditions, locomotion (open field) and sensory gating (prepulse inhibition, PPI). All studies used male Wistar rats. Pharmacokinetics after single dose of 2 mg/kg MDPV was measured over 6 h in serum, brain and lungs. The biotransformation study recorded 24 h urinary levels of MDPV and its metabolites after 4 mg/kg. The effect of 2 mg/kg and 4 mg/kg on body temperature (°C) was measured over 12 h in group- vs. individually-housed rats. In the open field, locomotion (cm) and its spatial distribution were assessed. In PPI, acoustic startle response (ASR), habituation, and PPI were measured (AVG amplitudes). In behavioural experiments, 1, 2, or 4 mg/kg MDPV was administered 15 or 60 min prior to testing. Thermoregulation and behavioural data were analysed using factorial analysis of variance (ANOVA). Peak concentrations of MDPV in sera, lung and brain tissue were reached in under 30 min. While negligible levels of metabolites were detected in tissues, the major metabolites in urine were demethylenyl-MDPV and demethylenyl-methyl-MDPV at levels three-four times higher than the parent drug. We also established a MDPV brain/serum ratio ~2 lasting for ~120 min, consistent with our behavioural observations of locomotor activation and disrupted spatial distribution of behaviour as well as moderate increases in body temperature (exacerbated in group-housed animals). Finally, 4 mg/kg induced stereotypy in the open field and transiently disrupted PPI. Our findings, along with previous research suggest that MDPV is rapidly absorbed, readily crosses the blood-brain barrier and is excreted primarily as metabolites. MDPV acts as a typical stimulant with modest hyperthermic and psychomimetic properties, consistent with a primarily dopaminergic mechanism of action. Since no specific signs of acute toxicity were observed, even at the highest doses used, clinical care and harm-reduction guidance should be in line with that available for other stimulants and cathinones.

## Introduction

### Background and context

“Novel psychoactive substances” (NPSs) are synthetic compounds that produce similar subjective psychological and behavioural effects to pre-existing illicit recreational drugs, however, they may circumvent legal controls owing to differences in their chemical structures [1]. In recent years, NPSs have proliferated across the globe [2, 3] as a result of their perceived “legality,” widespread availability [4] and of changes in socio-cultural attitudes where “sensible” recreational drug-taking has become increasingly normalised [5]. Unsurprisingly, rates of NPS dependence and toxic reactions/fatalities have risen simultaneously ([6, 7]) resulting in the increasing prominence of NPS (mis)use on public health agendas [8]. “Bath salts” is the colloquial street name for a class of NPSs where the principle psychoactive ingredient is a synthetic derivative of [9, 10]. Despite their common origin, cathinone derivatives have heterogeneous effects and mechanisms of action and it remains difficult to reliably predict the behavioural and physiological effects of NPSs based on their structural similarities [11]. Since controlled studies are not usually possible in humans, animal studies provide useful indicators of effects *in vivo* [12]. Studying effects (on an NPS-by-NPS basis) in animal models and *in vitro* continues to be a central approach oriented to obtaining knowledge with translational relevance to humans (e.g., harm reduction, clinical care). Here, we present data pertinent to further understanding of the acute pharmacokinetic, metabolic, behavioural, and thermoregulatory effects of the high-potency pyrovalerone cathinone 3,4-methylenedioxypyrovalerone (MDPV).

### Mechanisms of action

MDPV belongs to a sub-class of stimulating cathinones “pyrrolidinophenones” characterised by a complex chemical structure containing a pyrrolidine ring and an alkyl side-chain extending from the α-carbon [13]. Pyrrolidinophenones' stimulant effects reflect their inhibition of monoamine transporter sites where they are highly selective for the dopamine and norepinephrine transporter (DAT and NET), with particularly powerful effects on DAT, and only a negligible effect on the serotonin transporter (SERT) ([4, 14–20], but see [21]). To date, no evidence has been found (in rodents, or humans) that pyrrolidinophenones act as transporter substrate “releasers” [4, 14–16, 20] nor is there evidence that MDPV is active at non-transporter sites [16, 20]. Data from human embryonic kidney (HEK) cells expressing human transporters *in vitro* show a DAT/SERT inhibition ratio ranging from 100 to 300 [20, 22] and in rat brain synaptosomes, a ratio of 806 [18] for MDPV, values which far exceed those for other psychostimulants (e.g., amphetamine and cocaine), as well as most other pyrrolidinophenones [18, 20, 22]. MDPV is also much more effective at DAT than NET inhibition than other psychostimulants, e.g., for cocaine it is 50 times more potent at inhibiting DAT and ten times more potent at inhibiting NET [14, 18, 23, 24].

### Pharmacokinetics and metabolism

Recreational users report that the desirable subjective effects of MDPV include euphoria, sensory and physical stimulation [25, 26], similar to amphetamine (to which MDPV is chemically related; [1, 27], 7]). Recreational users typically ingest MDPV orally or by insufflation [4, 25]. Typical low-moderate active dosages for human recreational use range between 3 and 10 mg [25] with reported onset of subjective effects within 30 min and a duration of 120–210 min, with after-effects up to 8 h, [25, 28]. *In vivo* studies in rats that examined the concentrations of MDPV in plasma after subcutaneous (sc.) 0.5–3 mg/kg MDPV showed maximal plasma concentrations 10–20 min after administration, which then declined rapidly with an estimated elimination half-life of ~80 min [29, 30]. Only one previous study, however, has reported on the penetration of MDPV into the brain (after 1 mg/kg sc.), showing peak concentrations by 25 min in Sprague-Dawley rats [30].

*In vitro* studies of human liver microsomes have shown that MDPV is metabolised first to 3,4-dihydroxypyrovalerone (demethylenyl-MDPV) via *O*-demethylenation of its 3,4-methylenedioxy ring, and then to 4-hydroxy-3-methoxypyrovalerone (demethylenyl-methyl-MDPV) by *O*-methylation; these phase II metabolites are excreted in urine in conjugated form ([31, but see 32]). More recently it has been shown, that in rats, concentrations of demethylenyl-MDPV and demethylenyl-methyl-MDPV in plasma peak more slowly than MDPV, at around 3–4 h [29]. Demethylenyl-methyl-MDPV is the most abundant urinary metabolite in both species, humans and rats [32].

### Toxicity and thermoregulation

Human data on MDPV and pyrrolidinophenone toxicity is often confounded because “bath salts” commonly contain multiple cathinones and unknown contaminants [1, 7, 33]. However, since MDPV has been implicated more frequently in cases of serious intoxication [7, 34, 35] and confirmed in a number of fatalities [7, 36–39] than other cathinones, it may have higher risk of acute toxicity. Acute toxic effects of MDPV specifically involve over-stimulation of the cardiovascular system and the central nervous system (CNS), resulting in agitation, hyperthermia, and tachycardia [13, 26], and in one fatal case (where urine levels of MDPV were 670 ng/mL), coagulopathy, acidosis, rhabdomyolysis and anoxic brain injury were observed [38]. Psychiatric symptoms such as paranoia, delirium and hallucinations may be present, and serotonin syndrome, deterioration of muscle tissue and kidney failure have also been reported [26, 28, 37, 40–42]. Prominent in cathinone-related toxidrome (in humans) is hyperthermia; however, prior research on the effects of MDPV specifically on thermoregulation in rodents has been inconsistent. MDPV can elevate body temperature under conditions of higher ambient temperatures or social interaction, [21, 43, 44], however effects can be negligible, even under such conditions [45].

### Behavioural effects

MDPV is a highly reinforcing psychostimulant with notable addictive potential [20, 22, 46–51]. Increased extracellular dopamine (DA), particularly in the nucleus accumbens (NAcc), is an indicator of the reinforcement potential of drugs, and the accompanying locomotor response itself can be used as a marker of this. MDPV's stimulatory effects are most likely mediated by striatal extracellular DA ([29, 30], see [4]). MDPV has been shown to increase both locomotion in rodents ([14, 45, 52–55], see also [50], for a recent review) as well as increased NAcc DA [4, 14, 29]. Moreover, locomotor stimulation positively correlates with MDPV concentrations in the striatum [30], in plasma, and with extracellular DA concentrations [56] in the NAcc [4, 29]. The open field test is sensitive to stimulatory/sedative effects of centrally acting drugs, as well as having some sensitivity to emotionality (manifested as changes in the temporo-spatial characteristics of locomotor behaviour). Although effects of MDPV have been characterised in a number of other locomotor tests (activity cage, running wheel and rotorod), there is only one study of its effects in the open field using (Sprague-Dawley) rats, which showed 0.5 mg/kg intraperitoneally (ip.) increased locomotion. In mice, lower doses (1–3 mg/kg) MDPV increased locomotion and exploration were reported, with ataxia, hyperactivity and stereotypies emerging at doses 3–30 mg/kg [18, 43, 49, 53, 57]. Locomotor stimulation at lower doses and gross motor effects/stereotypies at higher doses are consistent with the inverted “U” curvilinear dose response identified for MDPV [45, 58].

Prepulse inhibition (PPI) of the acoustic startle response (ASR) is a behavioural operationalisation of sensorimotor gating that reflects pre-attentional filtering of redundant information [59]. PPI is useful in assessing the psychomimetic properties of drugs and is a behavioural endophenotype of psychosis [60]. Stimulants and cathinones can disrupt PPI, but usually only at higher doses ([61–68], but see [69]). As already described, MDPV use (in humans) can, like other cathinones, result in psychotic symptoms such as hallucinations, however to date, MDPV has not been tested in PPI, nor have any other pyrrolidinophenones.

### Aims and predictions

The aim of the present manuscript is to provide a detailed characterisation of the effects of subcutaneous (sc.) MDPV in the range 1–4 mg/kg with regard to pharmacokinetics (in sera, brain and lung), biotransformation (urine and sera), acute systemic toxicity (thermoregulation), locomotion (open field), and sensory gating (PPI). The study was performed as a part of an experimental series performed in our laboratory enabling direct comparisons with other NPSs belonging to cathinones, phenethylamines as well as aminoindanes (e.g., [66, 70, 71]). Based on these findings and methods as well as more recent ones presented by others [22, 31, 72], we expected MDPV to peak within first hour after administration in serum and brain, and due to its lipophilicity to accumulate into lung tissue. Additionally, levels of major metabolites were evaluated in tissues, sera as well as in urine. In order to simulate the typical environmental situations in which human MDPV use occurs, we measured body temperature under isolated and aggregated conditions with the prediction that aggregation will lead to more pronounced hyperthermic effects. In order to evaluate the characteristic inverted U locomotor dose-response curve we recorded open field activity at three MDPV doses at two testing-onsets following drug administration. Finally, we expected that MDPV, owing to its powerful effects on DAT, will disrupt PPI more potently than other ring-substituted cathinones tested in our laboratory.

## Method

### Design

For the pharmacokinetic study, samples of blood, whole brains and lungs were collected 30, 60, 120, 240, or 480 min after administration of 2 mg/kg MDPV. Concentrations of MDPV in sera and tissues were calculated as ng/ml or ng/g. The brain/serum ratio was calculated as mean brain concentration /mean serum concentration per sampling time point. The biotransformation study screened for MDPV metabolites and quantified MDPV and major metabolites in urine, sera (ng/mL) and tissues (ng/g) collected over 24 h after 4 mg/kg MDPV.

Open field and PPI experiments used a 4 × 3 factorial design with MDPV treatment (1, 2, 4 mg/kg or vehicle) and testing-onset (15 or 60 min) as independent factors. For the open field, trajectory length (cm, corrected for 3 cm deviations), thigmotaxis (measured as Σf_peripheral zones_/Σf_all zones_, where f = frequency of line crossings) and T_centre_ (calculated as Σ_time_ in the central zones) were measured. A repeated measures factor was also included in the open field experimental design in order to measure trajectory length over six 5 min time bins (4 × 3 × 6 mixed design). In the PPI experiment, all measures were derived from average startle amplitudes (AVG), and were as follows: % habituation (percentage reduction in ASR from six baseline trials, to the final six trials), ASR (mean ASR was derived from pulse alone trials) and % PPI (calculated as: [100–(mean prepulse–pulse trials/mean pulse alone trials)^*^100]) The thermoregulation study measured rectal temperature (°C) and used a 3 × 2 × 13 mixed factorial design with MDPV treatment (2, 4 mg/kg or VEH) and home-cage condition (group or individually caged) as independent factors and measurement time points as a repeated measures factor.

### Animals

Male outbred Wistar rats (Velaz, Czech Republic) weighing 180–250 g were housed in pairs at 22 ± 2°C on a 12/12 h light/dark cycle and with *ad libitum* water and standard diet. Rats were acclimatised for 7–10 days prior to testing, during which they were weighed twice and handled four times. All tests were conducted under standard conditions: humidity 30–70% and temperature 22 ± 2°C. All studies were carried out in accordance with the principles of laboratory animal care of the National Committee for the Care and Use of Laboratory Animals (Czech Republic), and according to Guidelines of the European Union (86/609/EU). The protocol was approved by the National Committee for the Care and Use of Laboratory Animals (Czech Republic) under the number: MEYSCR-27527/2012-31.

Across all experiments, 193 rats were used. For open field and PPI experiments, each had a total sample size of 60 (*n* = 10). Naïve rats were used for each experiment with the exception that (to reduce animal use) 40 rats from the open field were used for pharmacokinetic sampling (*n* = 8). For thermoregulation studies, *N* = 60 (*n* = 10). For the biotransformation study the total number of rats was three. As previously described, data from vehicle (VEH) control animals for PPI, open field and temperature studies were collected twice per annum as part of a series of standardised NPS studies undertaken in our laboratory (e.g., see [73]).

### MDPV doses

MDPV and other analytical standards were synthesised in-house at the Department of Organic Chemistry, University of Chemistry and Technology, Prague. In all studies, MDPV was dissolved in physiological saline in a volume of 2 ml/kg, and was always administered sc. as a single bolus. MDPV has been shown to be behaviourally active in locomotor tests in rodents at between 0.5 and 30 mg/kg across ip. and sc. routes. Based on previous behavioural research 1, 2, and 4 mg/kg doses were selected for behavioural testing with the expectation that 1 and 2 mg/kg would produce mild stimulatory effects similar to those sought by people. 4 mg/kg was selected to represent more extreme use, with stereotyped behaviours expected. Pharmacokinetic, biotransformation and thermoregulation studies tested 2 and/or 4 mg/kg were tested; these higher doses were chosen in order to increase the likelihood of detectable effects. The dose rage tested was similar to that used by Novellas et al. [30] where they argued that doses up to 3 mg/kg were within a range used by consumers.

### Pharmacokinetics: determination of MDPV in serum and tissues

After completing behavioural testing in the open field, rats were decapitated at 30, 60, 120, 240, or 480 min after administration of 2 mg/kg MDPV, and blood, whole brains and lungs were collected. Separated sera and tissues were stored at −20°C until the toxicological analyses. Sample preparation, LC and MS conditions, method of validation and calibration were based on Meyer et al. [32] and similar to other previously published methods (e.g., [31, 72]). For a full description of pharmacokinetic methods, please refer to Horsley et al. [73].

### Biotransformation study: determination of MDPV and its main metabolites in urine

Rats were administered 4 mg/kg MDPV, were placed individually into metabolic cages (Harvard Apparatus, USA) and their 24-h urine fractions were collected. During collection urine was maintained below 4°C throughout. Samples were subsequently stored at −40°C until analysis. The methods used to the screening of MDPV metabolites, and quantification of MDPV and its metabolites has been described more fully in Horsley et al. [73].

### Systemic toxicity: thermoregulation

Rats were housed singly vs. five to a home-cage and rectal temperature was measured for 10 s using a digital thermometer. The first three measurements were drug-free, and were taken hourly, 07:00–09:00 h (inclusive) whereupon 2, 4 mg/kg MDPV or VEH was administered. Thereafter, observations were at 0.5 h intervals 09.30–11.00 h, before resuming hourly measurements 12.00–17.00 h. For a fuller description, see e.g., Páleníček et al. [70, 71].

### Behaviour: open field and PPI

#### Open field

Rats were administered MDPV at 1, 2, 4 mg/kg or VEH, and then 15 or 60 min post-administration they were placed individually into the centre of the open field apparatus (a 68 × 68 × 30 cm square black plastic arena) and their behaviour was video-recorded for 30 min. Ethovision Colour-Pro version 3.1.1, (Noldus, Netherlands) was used for behavioural capture and pre-processing. During pre-processing, the arena was virtually divided into 5 × 5 identical square zones with 16 located around the periphery and 9 centrally in order to derive thigmotaxis and T_centre_ variables (see section Design for calculations). The procedures used were the same as those used previously, e.g., Páleníček et al. [70, 71].

#### PPI

Two days before test, rats were acclimatised to the startle chamber (SR-LAB, San Diego Instruments, California, USA) with a drug-free five min pre-exposure to five pulse alone stimuli (115 dB/20 ms) over 75 dB continuous white noise. On the test day, 15 or 60 min prior to testing, rats were administered 1, 2, 4 mg/kg MDPV or VEH, placed into the startle chamber and acclimatised for five min to a continuous 75 dB white noise. They were then presented with six 125 dB/40 ms duration pulse alone trials, followed by 60 pseudorandomised trials of the following: (A) pulse alone: 40 ms 125 dB; (B) prepulse-pulse: 20 ms 83 dB or 91 dB prepulse, a variable (30, 60 or 120 ms) inter-stimulus interval (ISI: mean 70 ms), then 40 ms 125 dB pulse; (C) 60 ms no stimulus. Finally, six pulse alone trials were delivered. There were 72 trials in total with inter-trial intervals (ITIs) of 4– 20 s (mean ITI = 12.27 s). A fuller description can be found in our previously published work (e.g., [70, 71]).

### Statistics

Statistical analyses of behavioural and thermoregulation data were conducted using IBM SPSS version 22. Unless stated otherwise, default alpha was set at *p* = 0.05, and tests were two tailed. For open field, PPI, and temperature analyses we used factorial analysis of variance (ANOVA). For repeated measured ANOVAs, where Mauchly's test of sphericity was significant Greenhouse-Geisser corrections were used. Planned pairwise comparisons (to follow up significant main effects and interactions involving MDPV) used independent *t*-tests. In order to limit inflation of type 1 error the number of comparisons was restricted to those necessary to test the primary hypotheses (MDPV vs. VEH, no between dose comparisons were made), and in the thermoregulation study, comparisons were focused on early time points with observable mean differences. Where Levene's test for equality of variance was significant, corrected statistics are presented. Corrected degrees of freedom are rounded to the nearest whole number for presentational purposes.

## Results

### Pharmacokinetics, metabolism, and hyperthermic response

#### MDPV in serum and tissue after 2 mg/kg MDPV

**Figure 1:**
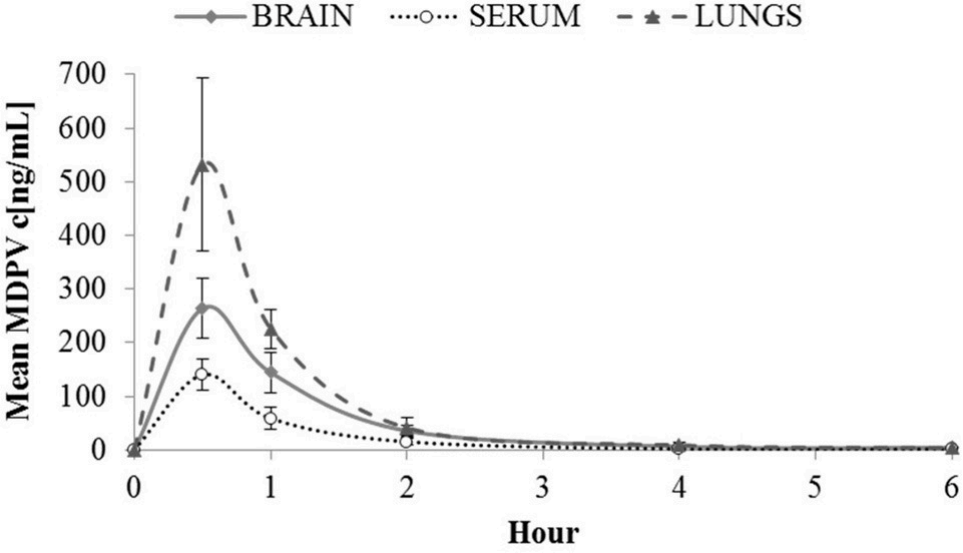
Mean MDPV concentrations (c[ng/mL]) in brain, serum and lungs observed over 6 h at the following time points 0.5, 1, 2, 4, and 6 h after sc. administration of 2 mg/kg of MDPV. At 6 h, all tissue values were <4. For graphical purposes, at 6 h a mean of 4 and a standard deviation of 1 is used. Likewise at 6 h, all serum values were <1 (so a mean of 1, and standard deviation of 0.25 are used). Error bars show ±1 standard deviation.

Maximal MDPV (Figure 1) concentrations in sera were likely attained before the first measurement, since we saw the highest levels at 30 min (140 ng/mL). The same applied to tissues where influx was not detectably delayed compared to serum since maximal concentrations were also observed at 30 min: 263.97 ng/g in brain, and 531.58 ng/g in lung. Until 2 h, the brain/serum ratio was between 1.8 and 2.5, and at 4–6 h the brain/serum ratio was ~4 however, by this time levels in serum and tissue were very low, and almost undetectable by 6 h.

#### MDPV and metabolites in urine, sera, and tissues after 4 mg/kg MDPV

**Figure 2:**
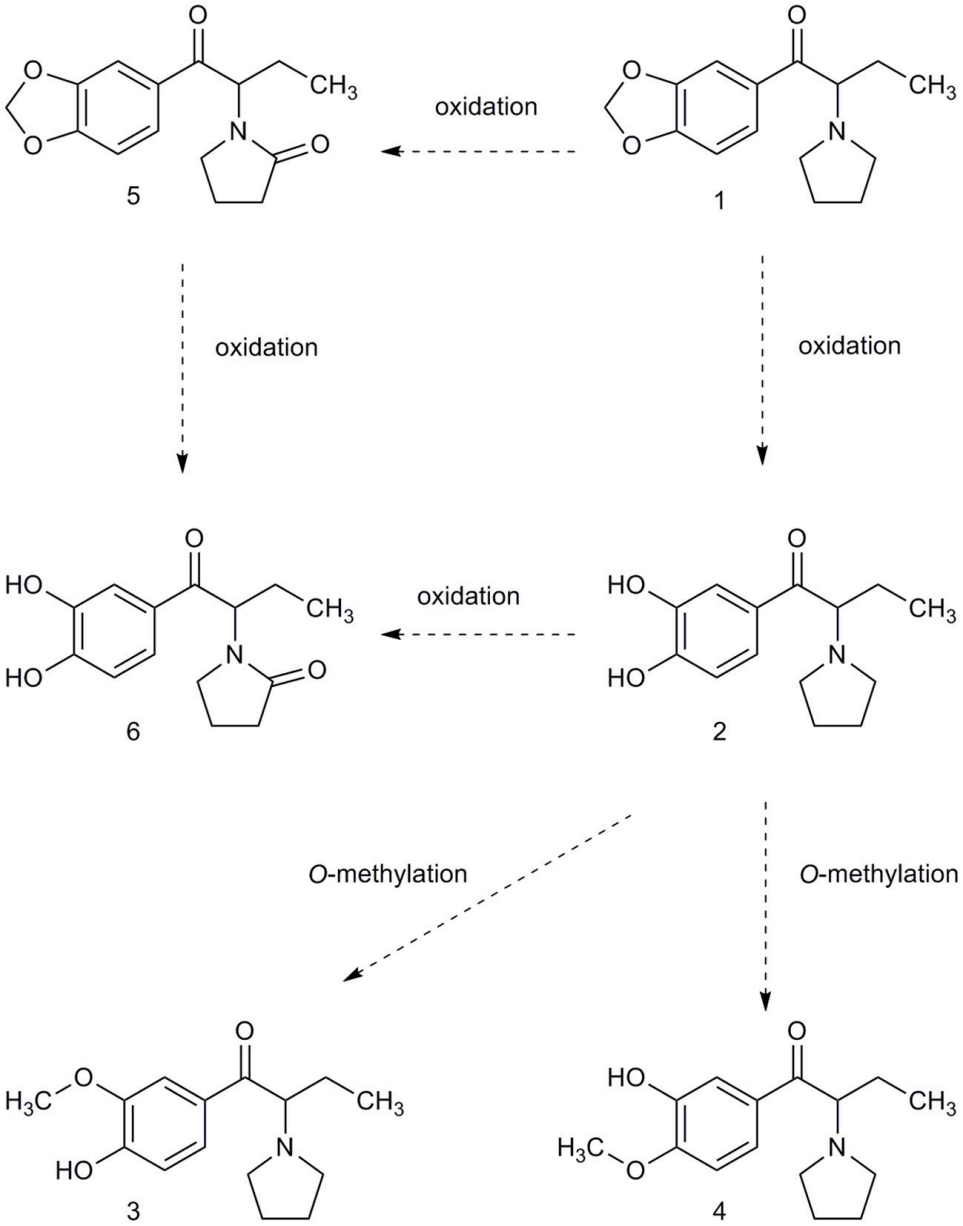
The metabolites of phase I were determined using LC-MS/MS and synthetized metabolite standards. Phase II metabolites of compound (2) and (6) were confirmed using UHPLC-QTOF(MS) as glucuronide demethylenyl-methyl-MDPV and glucuronide demethylenyl-MDPV.

MDPV itself was present in urine but was extracted primarily as metabolites. We adopted the nomenclature of Meyer et al. [32] for the most abundant substances detected which were 4-hydroxy-3-methoxypyrovalerone (demethylenyl-methyl-MDPV), followed by 3,4-dihydroxypyrovalerone (demethylenyl-MDPV) (see Table 1 and Figure 2 for quantification and structures). All other metabolites were in a minority.

**Table 1:** The urinary metabolites of phase II were screened using UHPLC-QTOF(MS) and the following glucuronides were confirmed: glucuronide demethylenyl-methyl-MDPV and glucuronide demethylenyl-MDPV.

	**Concentration in urine (ng/mL)**		
	**Demethylenyl-MDPV**	**Demethylenyl-methyl-MDPV**	**MDPV**
Urine 1	704	398	122
Urine 2	586	790	227
Urine 3	284	145	74

In tissues, the metabolites demethylenyl-MDPV, demethylenyl-methyl-MDPV, demethylenyl-oxo-MDPV, and oxo-MDPV) were present at very low levels. A maximum value of 8 ng/ml demethylenyl-methyl-MDPV in the brain was observed, and 27 ng/ml in the lungs). In serum demethylenyl-oxo-MDPV was present at low levels (maximum value 23.8 ng/ml), and oxo-MDPV was almost undetectable (maximum value 2.8 ng/ml).

#### Rectal temperature after 2 or 4 mg/kg MDPV

**Figure 3:**
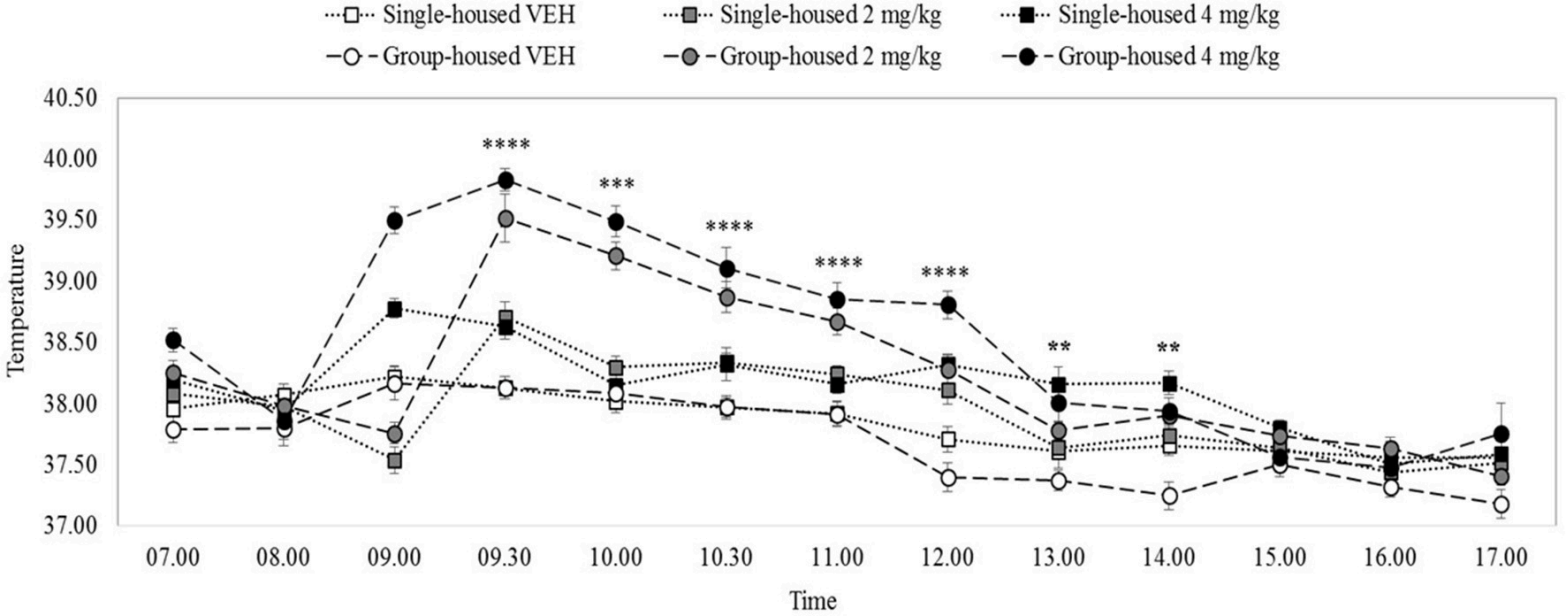
Mean rectal temperature (°C) over 10 h in rats (*n* = 10) housed individually (solid lines) or in groups of five (dashed lines). 4 mg/kg MDPV (black markers), 2 mg/kg MDPV (grey markers) or VEH (white markers) was administered sc. at 09.00 (black dotted vertical line). Error bars show ±1 standard error of the mean. Asterisks indicate significant differences from VEH at minimum *p* < 0.05.

There were significant main effects on rectal temperature of drug and time, minimum *F*_(2, 54)_ = 44.08, *p* = 0.0001, but no main effect of home-cage, *F*_(1, 54)_ = 0.19, *p* = 0.66. All interactions, including the three-way drug x home-cage x time interaction were significant, minimum *F*_(7, 383)_ = 2.79, *p* = 0.001. Figure 3 shows that the groups had equivalent body temperatures at the 8.00 h baseline temperature measurement, maximum *t*_(38)_ = 0.41, *p* = 0.68. VEH rats' temperatures remained steady at about 37.7–38°C, throughout the temporal observation, irrespective of housing condition.

After 2 or 4 mg/kg temperature increased in both individually- and (more dramatically in) group-housed rats, peaking at 9.30 h and then diminishing. After 2 mg/kg, temperature was significantly elevated in group- and individually-housed rats until 12.00 noon, minimum *t*_(18)_ = 2.32, *p* = 0.03 (with some fluctuating differences thereafter). Compared to 2 mg/kg the increase in temperature after 4 mg/kg was longer-lasting: significant elevations were seen in group- and individually-housed rats at all-time measurements up until 14.00 h (except 10.00 h, individually-housed), minimum *t*_(18)_ = 1.86, *p* = 0.04, one tailed (with some fluctuating differences thereafter).

### Behaviour: open field and PPI

#### Locomotor stimulation in the open field

The main effect of testing-onset and the bins x testing-onset interaction were not significant, maximum *F*_(3, 183)_ = 2.12, *p* = 0.11. All other main effects and interactions, including the three-way drug × testing-onset × bins interaction were significant, minimum *F*_(8, 183)_ = 3.23, *p* = 0.001.

**Figure 4:**
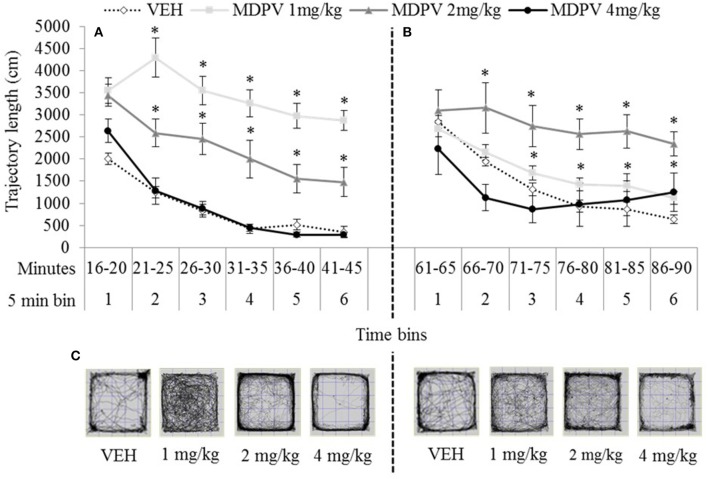
Mean trajectory length (cm/5 min over 30 min) tested 15 min **(A)** or 60 min **(B)** after drug administration: sc. MDPV 1 mg/kg (light grey), 2 mg/kg (mid-grey) or 4 mg/kg (black) vs. VEH (white). Shown alongside 5 min bins (on the x axis) are minutes elapsed since drug administration. Error bars show ±1 standard error of the mean. Asterisks indicate significant differences from VEH for the 1 and 2 mg/kg groups, at minimum *p* < 0.05. Example trajectory patterns are shown in **(C)**.

At both 15 and 60 min testing-onsets the 4 mg/kg treated rats were indistinguishable from VEH (except in bin 1, 60 min), maximum *t*_(13)_ = 1.69, *p* = 0.11). At both testing-onsets, rats showed a normal pattern of locomotor habituation (progressively diminishing activity over the session) which was not disturbed by any dose MDPV. However, 1 and 2 mg/kg treated rats were significantly more active than VEH at both the 15 min testing-onset, minimum *t*_(11)_ = 2.98, *p* = 0.00 (Figure 4A), and at the 60 min testing-onset after 1 mg/kg (bins 4–6: minimum *t*_(18)_ = 2.54, *p* = 0.02), and after 2 mg/kg (bins 2–5: minimum *t*_(18)_ = 2.11, *p* = 0.05 (Figure 4B).

#### Spatial distribution of locomotor behaviour in the open field

**Figure 5:**
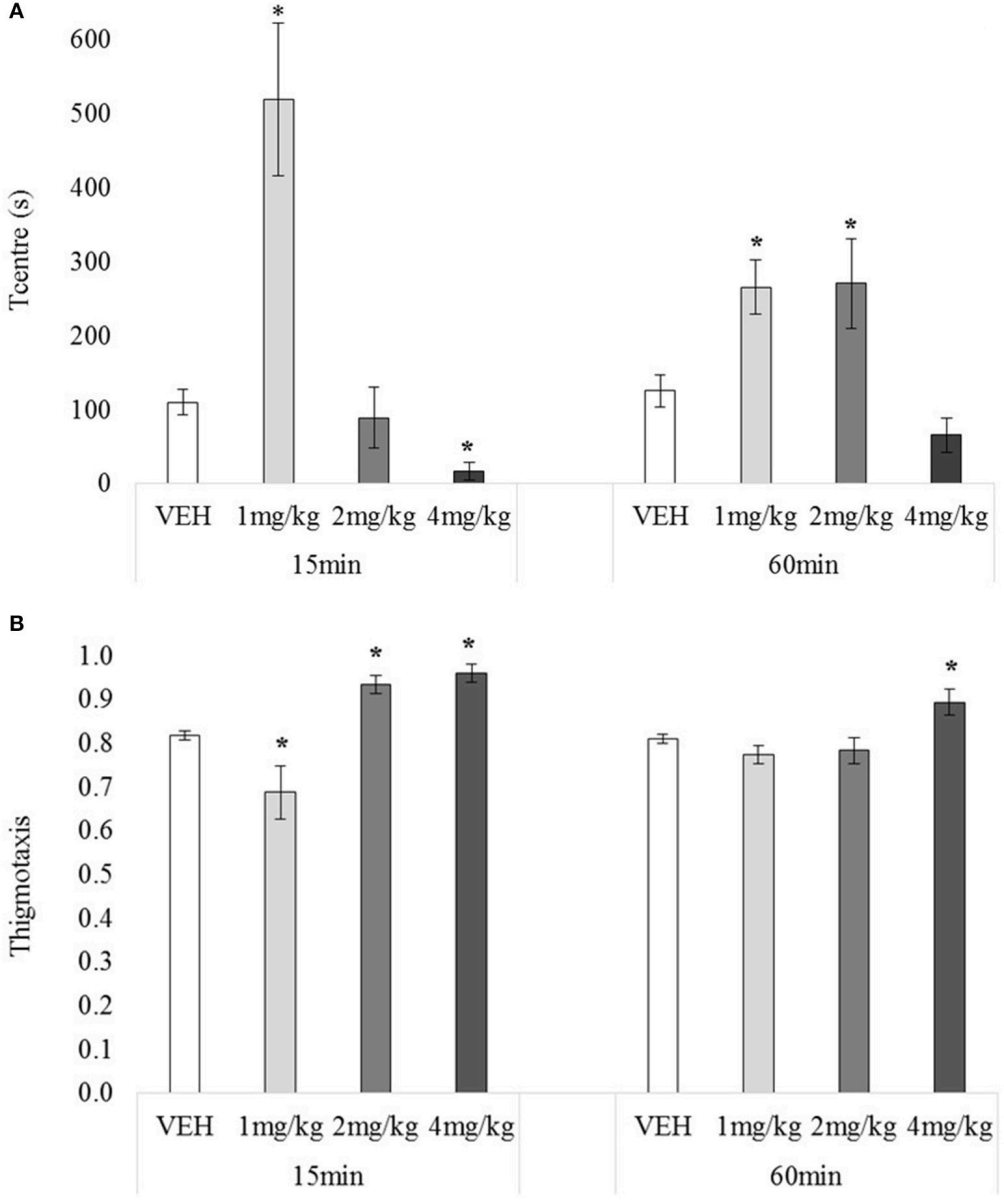
Mean T_centre_ **(A)** and mean thigmotaxis (**B**) over 30 min tested 15 or 60 min after drug administration: sc. MDPV 1 mg/kg (light grey), 2 mg/kg (mid-grey) or 4 mg/kg (dark grey) vs. VEH (white). Error bars show ±1 standard error of the mean. Asterisks indicate significant differences from VEH at minimum *p* < 0.05.

There was no main effect of testing-onset on T_centre_, *F*_(1, 72)_ = 0.00, *p* = 0.95, but the main effect for drug, and drug x testing-onset interaction were significant, minimum *F*_(3, 72)_ = 7.13, *p* = 0.00, Figure 5A. At 15 min 1 mg/kg-treated rats spent significantly more time in the centre (than VEH), and 4 mg/kg –treated rat significantly less, minimum *t*_(10)_ = 3.92, *p* = 0.00, with 2 mg/kg having no significant effect, *t*_(12)_ = 0.47, *p* = 0.64. At 60 min, 1 and 2 mg/kg increased time in the centre, minimum *t*_(18)_ = 2.26, *p* = 0.04, with 4 mg/kg having no effect, *t*_(18)_ = 1.87, *p* = 0.07.

There was no main effect of testing-onset on thigmotaxis, *F*_(1, 72)_ = 2.76, *p* = 0.10, however there was a significant main effect of drug, and drug x testing-onset interaction, minimum *F*_(3, 72)_ = 5.77, *p* = 0.00, Figure 5B. At 15 min, 2 and 4 mg/kg increased, and 1 mg/kg decreased thigmotaxis, minimum *t*_(10)_ = 2.27, *p* = 0.05. At 60 min, 4 mg/kg again increased thigmotaxis, *t*_(18)_ = 2.55, *p* = 0.02, but 1 and 2 mg/kg had no significant effect, maximum *t*_(18)_ = 0.77, *p* = 0.45.

#### Prepulse inhibition

**Figure 6:**
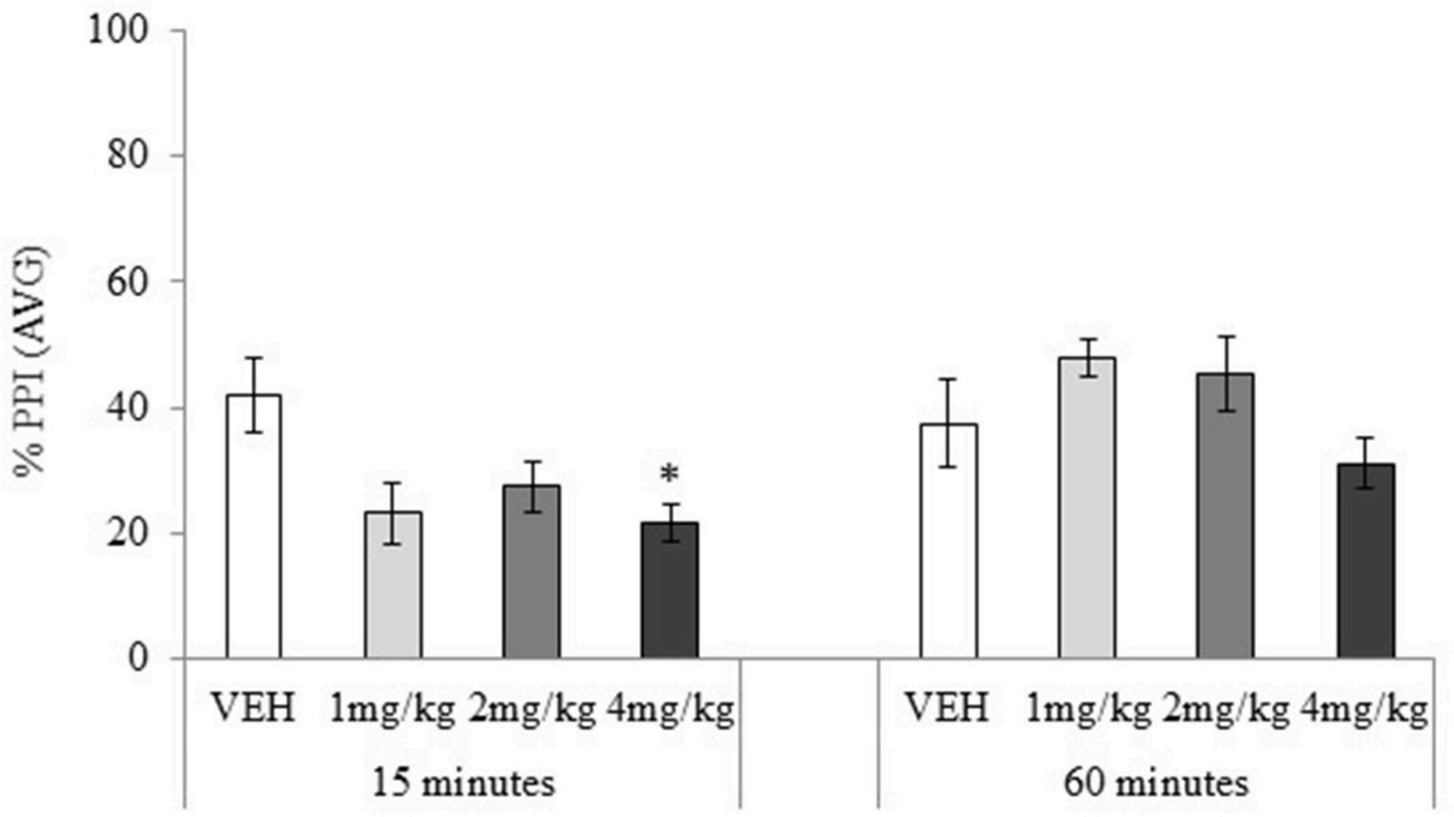
Mean percentage (%) prepulse inhibition (AVG amplitude) tested 15 or 60 min after drug administration: sc. MDPV 1 mg/kg (light grey), 2 mg/kg (mid-grey) or 4 mg/kg (dark grey) vs. VEH (white). Error bars show ±1 standard error of the mean. Asterisks indicate significant differences from VEH at minimum *p* < 0.05.

Habituation and ASR data showed no significant main effects or interactions, maximum *F*_(3, 72)_ = 2.25, *p* = 0.24. PPI data showed no main effect of the drug treatment, *F*_(3, 72)_ = 2.21, *p* = 0.09, but the main effect of testing-onset, and the drug treatment × testing-onset interaction were significant, minimum *F*_(3, 72)_ = 2.73, *p* = 0.05, Figure 6. In the 15 min testing-onset group, 4 mg/kg significantly disrupted PPI, *t*_(18)_ = 2.70, *p* = 0.02, and 1 and 2 mg/kg had a marginally significant effect, maximum *t*_(18)_ = 1.95, *p* = 0.07. At 60 min testing-onset, none of the MDPV doses significantly affected PPI, maximum *t*_(18)_ = 1.67, *p* = 0.11.

## Discussion

As expected, our pharmacokinetic findings were in line with previous results for serum and brain, and we reported new data on lungs; after 2 mg/kg, MDPV reaches its peak concentrations in serum and tissues in under 30 min and declines rapidly thereafter. Our biotransformation study detected a number of metabolites in sera and tissues, however at very low levels. In urine, concentrations of metabolites exceeded those of MDPV itself, and we confirmed that demethylenyl-methyl-MDPV the most abundant. As previous studies have shown we found a small increase in temperature in individually-housed rats, which was exacerbated by crowded cage conditions. In line with existing findings, our behavioural effects were systematically related to drug concentrations/dose, and showed good correspondence with the known pharmacokinetic time-course for MDPV. As expected, in the open field, lower doses generally stimulated locomotion and exploration, whereas the higher dose initially induced stereotypy/gross suppression of motor behaviour, consistent with stimulant-typical psychomimesis. We presented additional original data which supported this: 4 mg/kg can induce transient psychomimesis shown as disrupted PPI.

### Pharmacokinetics and metabolism

The highest concentrations of MDPV in serum, brain, and lung tissue were recorded at 30 min followed by a rapid decline, suggesting that in all cases, the actual peaks were prior to our first measurement. Previous pharmacokinetic data in rats (which were not published at the time of our data collection) showed peak plasma concentrations of MDPV at 10–20 min post-administration [29, 30]. It is therefore almost certain that our value of 140 ng/mL for peak serum concentration is an underestimation. Even so, concentrations in this range in the plasma of human MDPV users are more typical of cases of acute intoxication and overdose (in excess of 50–300 ng/mL) than of recreational use where milder effects are associated with much lower blood levels at ~10– 50 ng/mL [74].

Novellas et al. [30] reported that MDPV reached the striatum of the brain around 5 min after sc. MDPV (1 mg/kg) in rats and peaked 20–25 min after that, suggesting that our observed peak brain concentration value at 30 min (263.97 ng/g) was likely close to the actual value. From 30 min and the following 2 h, the brain concentration was approximately twice that in the sera. This is consistent with the brain/serum ratio of 2.21 reported by Novellas et al. [30], and reflects MDPV's lipid solubility and capacity to cross the blood-brain barrier. Evidence suggests a kinetic profile of fast onset and relatively short duration, consistent with our short-lasting peak behavioural effects, and with estimates of the onset and duration of subjective effects in humans [25, 28]. MDPV's short duration may have a causal role in re-dosing and escalating use (shown in human and rodent studies), and together with fast penetration of the brain [30] these characteristics are indicative of MDPV's addictive potential [36].

We observed levels of MDPV that were approximately four times higher in lungs than in sera. Rapid transition of substances (such as drugs) from blood to lungs is characteristic of parenterally administered cationic compounds with a lipophilic profile [75]. The rapidity and magnitude of accumulation is partly a consequence of the relatively large volume of blood flowing to the lungs in compared to other organs, such as the brain, where molecules become trapped by lysosomes and mitochondria and accumulate in lung tissue as gradually eluting pools [75]. In consequence, accumulation in, and elimination from lung tissue is usually rapid, which was supported by our data, where lung concentrations of MDPV declined more quickly than in the brain. The relevance (if any) of drug accumulation in the lungs for MDPV toxicity is unclear; pyrrolidinophenone toxicity primarily involve CNS and cardiovascular related problems [13, 56].

As expected, MDPV itself was detected in urine at only very low concentrations, instead MDPV was detected as two major metabolites demethylenyl-MDPV and demethylenyl-methyl-MDPV. These urinary metabolites were at concentrations approximately three to four times higher than the parent drug. Other metabolites were detected in urine, serum, brain and lung but again at very low concentrations. Taken together with pharmacokinetic findings here and previous studies [30–32, 72], MDPV appears to be cleared from sera and tissues relatively rapidly (here almost undetectable by 6 h), and by 24 h only very small amounts of MDPV in the form of metabolites remain in the body the majority having been excreted in urine. Toxicological tests that seek to confirm MDPV in blood or tissues after 6 h should search for a range of MDPV metabolites rather than MDPV itself and use the most sensitive methods available. Our sample size for the biotransformation study was small, and there was variability in the data, both of which may have contributed to the observation of lower concentrations, and few metabolites than other studies have shown previously.

### Thermoregulation

As in our pharmacokinetic study, we found peak temperatures at our earliest measurement time-point (30 min post-administration), therefore, we cannot exclude the possibility that temperatures were increased prior to this. MDPV increased body temperature for several hours up to a maximum of ~0.5°C in rats housed individually at ~22°C. This small increase is in agreement with previous work using individually-housed Wistar rats where MDPV's effects on body temperature (at a similar dose range) were negligible even at 30°C ambient temperatures [45]. A number of findings have been published now that demonstrate that environmental crowding and hotter ambient temperatures can exacerbate drug-induced hyperthermia [70, 73, 76, 77]. We found longer-lasting increases in temperature in group-housed rats, up to 2°C. MDPV increases brain and body temperature in mice at high ambient temperatures and under conditions of social interaction [21, 43, 44], perhaps due to combined DAT-mediated hyperactivity and NET-mediated sympathomimetic effects [20, 22], although recent research has also implicated 5-HT [21]. If environmental/social conditions can potentiate both locomotor and temperature effects simultaneously, these circumstances might increase the risk of toxic reactions. On the other hand, although MDPV toxicity and fatalities in humans have been documented [7, 34–39, 41], they remain relatively uncommon given the prevalence of cathinone use. Moreover, the doses tested here equate to very high human doses (in excess of ~20 mg, [78]), that are in the range where adverse reactions would be expected [25].

### Locomotor behaviour

As expected, the locomotor stimulant action of MDPV across the three doses (1, 2, and 4 mg/kg) was consistent with the inverted “U” behavioural dose-response of dopaminergic agonists (such that progressively larger doses resulted in progressively increasing, then decreasing activity). In our behavioural studies, the 15 min testing-onset synchronised with peak serum and brain concentrations as well as their initial decline. Effects over testing-onsets showed that locomotor effects related to declining systemic levels of MDPV, e.g., effects of 1 mg/kg at 15 min were equivalent to residual effects of 2 mg/kg at 60 min. Other pyrrolidine-containing synthetic cathinone derivatives, α-pyrrolidinopropiobutiophenone (α-PBP), and α-pyrrolidinopentiophenone (α-PVP), as well as MDPV, show this inverted “U” curvilinear relationship with locomotor stimulation in rats ([14, 45, 52, 58] but see [53] for effects in mice). The normal pattern of locomotor habituation was not disturbed by lower doses of MDPV, however at 4 mg/kg (which induced some hypolocomotion at 15 min) by 80–90 min post-drug administration, locomotion appeared to be increasing. Had testing continued, hyperlocomotion may have been observed as brain drug concentrations diminished, as previously reported [45, 52]. MDPV's potent stimulatory effects most likely relate to its potent DAT inhibition, coupled with its lack of capacity to increase extracellular 5-HT which can attenuate DA-induced locomotor effects [4].

Rats usually avoid the aversive centre of the open field (which is brighter more and open) preferring to spend time in the periphery (next to the arena walls). The spatial characteristics of locomotor behaviour in this paradigm can provide some indication of drug effects on emotionality with increased exploration of the centre suggesting decreased emotionality (anxiolytic-like effects) and *vice versa*. [79–81]. In our experience, effects on T_centre_ and thigmotaxis are usually (but not always) the inverse of one another, however, whilst related, T_centre_ may be more sensitive to effects on emotionality, and thigmotaxis to locomotor stereotypy. MDPV has been shown previously to increase exploration in mice [53], as well as induce stereotypy (at a range of doses) in rats and mice [30, 45, 53, 72]. Here, as the dose/systemic levels of MDPV declined below ~2 mg/kg there was increased exploration of the centre (unconfounded by thigmotaxis), possibly indicative of anxiolytic effects (perhaps equating to positive affect or euphoria in humans at acute lower doses; [25]). At ~2 mg/kg or more, there was only increased thigmotaxis indicative of locomotor activation and/or emerging stereotypy. Stimulant-typical stereotypies emerge in a systematic manner [30, 72, 82], which was shown here, and, with MDPV, are reversible with the typical antipsychotic (a DA D2 antagonist), haloperidol (e.g., [30]), which is indicative of its DA-ergic origin and psychomimetic potential.

### Sensorimotor gating and psychomimesis (PPI)

Only 4 mg/kg MDPV disrupted PPI significantly and only transiently so (at the 15 min testing-onset), when plasma levels of MDPV were at their peak, and peak brain concentration was developing. By 60 min, MDPV levels in sera and brain had declined by ~30–40% to around 50 and 150 ng/mL respectively, and PPI was no longer significantly disturbed. A specific disruption of PPI (i.e., in the absence of significant baseline drug effects on habituation or ASR that might confound interpretation) reflects dysfunctional sensorimotor gating, and is an index of the psychomimetic properties of drugs [60]. Evidence to date has shown that other (ring-substituted) cathinones and stimulants can disrupt PPI, but usually only at higher doses [61–69]. Although different cathinones and stimulants operate via different DA-ergic mechanisms (i.e., DAT inhibition and/or non-exocytotic efflux) the net result is an increase extracellular DA. 5-HT-ergic compounds more reliably disturb PPI (e.g., [73, 83, 84]), however it would appear that hyperdopaminergia alone is sufficient to disrupt PPI, since MDPV has negligible effects on 5-HT [14, 85]. Moreover, cathinone-induced PPI deficits and stimulant-typical psychomimesis can be reversed with typical and atypical antipsychotics [30, 86] which have DA D2 receptor antagonism in common [87], suggesting a role for this receptor subtype specifically [63]. Our findings indicate that the duration of psychological symptoms is likely to be short, which suggests that in cases of acute MDPV intoxication, adverse psychological effects may pass relatively quickly.

## Conclusions

In conclusion, we confirmed MDPV's typical DA-ergic psychostimulant drug profile: at lower doses, it modestly increased locomotion and exploration; at higher doses it produced stimulant-typical psychomimetic and gross motor effects/stereotypy. Behavioural findings have relevance to recreational users who, in striving for stimulatory effects may take large amounts of MDPV, unaware that this will only delay rather than increase stimulation (which is modest, in any case) and induce adverse psychological and physical effects instead, with risks of toxicity (especially if taken in combination with other drugs) increasing with dose. However, our lowest dose of MDPV, 1 mg/kg, has been calculated as equivalent to 10 mg human dose and our highest dose is equivalent to 30 mg or more [78]. Thus even our lowest dose, whilst within the range used by people, was nevertheless a high dose, in the range associated with unwanted effects [25]. Responsible and occasional use of sensible doses of MDPV alone is probably relatively low-risk for most healthy people. However, users should be aware that MDPV's acute effects may create interactions between their activity levels, environmental and social circumstances and potentiate temperature dysregulation. Moreover, it is important, in a world where NPSs proliferate [3, 36] and where “sensible” recreational drug-taking is increasingly normalised [4, 5] that even where acute use appears low risk, the possible effects of long-term, chronic or binge use of MDPV on psychological and physical health are kept in mind. These are simply, as yet, unknown, and data will only emerge in years to come. The short-lasting behavioural effects and pharmacokinetics shown here are consistent with existing evidence that MDPV has characteristics that encourage risky use (such as re-dosing, binge or poly-drug use, 36) as well as significant addictive potential.

## Author contributions

All authors made a substantial contribution to the conception or design of the work; or the acquisition, analysis, or interpretation of data for the work. All authors were involved in drafting the work or revising it critically for important intellectual contents. All authors gave final approval for the current version of the work to be published. All authors agree to be accountable for all aspects of the work in ensuring that questions related to the accuracy or integrity of any part of the work are appropriately investigated and resolved.

### Conflict of interest statement

The authors declare that the research was conducted in the absence of any commercial or financial relationships that could be construed as a potential conflict of interest.
